# Postoperative paralytic ileus after cytoreductive surgery combined with heated intraperitoneal chemotherapy

**DOI:** 10.1515/pp-2019-0026

**Published:** 2019-11-12

**Authors:** Jesper Nors, Jonas Amstrup Funder, David Richard Swain, Victor Jilbert Verwaal, Tom Cecil, Søren Laurberg, Brendan John Moran

**Affiliations:** Department of Surgery, Aarhus University Hospital Skejby, Aarhus N, Denmark; Peritoneal Malignancy Unit, Hampshire Hospitals NHS Foundation Trust, Basingstoke, UK; Peritoneal Malignancy Institute Basingstoke, Basingstoke, UK

**Keywords:** cytoreductive surgery, HIPEC, peritoneal carcinomatosis, postoperative ileus, treatment outcome

## Abstract

**Background:**

Patients with peritoneal malignancy treated by cytoreductive surgery (CRS) combined with hyperthermic intraperitoneal chemotherapy (HIPEC) are prone to develop postoperative paralytic ileus (POI). POI is associated with significant increase in both morbidity and mortality. CRS and HIPEC commonly result in prolonged POI (PPOI). The objective was to clarify the extent of PPOI in patients treated by CRS and HIPEC for peritoneal malignancy.

**Methods:**

This was a prospective multicenter study including patients operated with CRS and HIPEC at the Department of Surgery, Aarhus University Hospital, Denmark and the Peritoneal Malignancy Institute, Basingstoke, United Kingdom. A total of 85 patients were included over 5 months. Patients prospectively reported parameters of postoperative gastrointestinal function in a diary from post-operative day 1 (POD1) until discharge. PPOI was defined as first defecation on POD6 or later.

**Results:**

Median time to first flatus passage was 4 days (range 1–12). Median time to first defecation was 6 days (1–14). Median time to removal of nasojejunal tube was 4 days (3–13) and 7 days (1–43) for nasogastric tube. Forty-six patients (54%) developed PPOI. Patients with PPOI had longer time to first flatus (p<0.0001) and longer time to removal of nasojejunal tube (p=0.001). Duration of surgery correlated to time to first flatus (p=0.015) and time to removal of nasogastric or nasojejunal tube (p<0.0001) but not to time to first defecation (p=0.321).

**Conclusions:**

Postoperative gastrointestinal paralysis remains a common and serious problem in patients treated with CRS and HIPEC.

## Introduction

The aim of cytoreductive surgery (CRS) is to remove all macroscopic malignant tumor and combine the surgery with intra-operative hyperthermic intraperitoneal chemotherapy (HIPEC) [1, 2]. Combined CRS and HIPEC is a treatment with curative intent with an increasing interest and usage worldwide and promising long-term results in selected patients with peritoneal malignancy (PM) [3, 4, 5, 6, 7].

CRS involves extensive intraabdominal surgery rendering patients prone to develop paralytic postoperative ileus (POI) [8, 9, 10, 11, 12, 13]. POI is a complex patho-physiological postoperative condition characterized by reduced, or absent bowel movement, and delayed gastric emptying. POI results in a range of symptoms including inability to tolerate enteral nutrition, nausea, abdominal distension, and non-passage of flatus or stools [8, 9, 10, 11, 12]. POI is also associated with increased morbidity, a prolonged hospital stay and increased mortality [9, 14, 15, 16].

The incidence and length of POI following colorectal surgery has reduced mainly by introduction and widespread use of the ERAS (Enhanced Recovery After Surgery) program [17, 18, 19, 20]. The shift from open to laparoscopic surgery is also likely to have been a significant factor in a reduction in the incidence of POI [12, 19, 20]. However, open surgery is still required in a number of patients, including those with advanced disease or recurrent cancer and in patients with peritoneal metastases [21]. In all patients after abdominal surgery, but particularly those who have had major open abdominal surgery, POI can develop into prolonged POI (PPOI), defined as more than 5 days without gastrointestinal function [8, 11, 18, 22, 23]. The consequences of PPOI include delayed enteral feeding, lowered immune function, and increased readmission rates [8, 9, 10, 11, 12, 20, 22, 24]. PPOI is frequent following extensive oncological surgery, yet little has been published on the topic [23].

The primary aim of this study was to describe the incidence and duration of PPOI after advanced abdominal cancer surgery for peritoneal malignancy, treated by CRS and HIPEC when applying contemporary postoperative regimes of the ERAS protocol [12, 19].

## Materials and methods

### Patients

This a prospective study in patients with peritoneal malignancy, treated by CRS and HIPEC at the Peritoneal Malignancy Institute, Basingstoke, United Kingdom, and the Department of Surgery, Aarhus University Hospital, Aarhus, Denmark. Both centers included patients treated during a 5 months study period (Basingstoke: October–November 2016 and February–April 2017, Aarhus; February–June 2017). All patients had CRS and HIPEC for either pseudomyxoma peritonei (PMP), colorectal cancer peritoneal metastases (CPM), appendix cancers including goblet cell carcinoid tumors, ovarian cancer or peritoneal mesothelioma. Inclusion in the study was voluntary and required informed consent.

Patients with extensive extraperitoneal disease, age>75 years, or an American Society of Anesthesiologists (ASA) scoreIII were excluded. Six or seven regions estimated by the Simplified Peritoneal Cancer (SPC) Score [21] led to exclusion for all diseases except PMP where more extensive disease was allowed.

Baseline characteristics and clinical parameters were registered prospectively (gender, body mass index (BMI), preoperative ASA score). BMI was calculated using the World Health Organization definition; weight (kg)/height squared (m^2^). The extent of peritoneal disease was evaluated and documented in all patients using the Peritoneal Cancer Index-score (PCI) [25].

### Perioperative management in Basingstoke

All patients underwent a CT of the thorax, abdomen and pelvis, unless already performed within the last 6 months for PMP or 3 months for CPM or other pathology. Selected patients were evaluated by a diagnostic laparoscopy to clarify the extent of peritoneal malignancy or to obtain a biopsy. All patients were evaluated by a multidisciplinary team prior to CRS and HIPEC. All patients had mechanical bowel preparation. Patients had a liquid diet for 24 h prior to surgery and were fasted overnight or for 6 h prior to induction of anesthesia). All patients had compression stockings and prophylactic thromboembolic treatment in the form of low-molecular-weight heparin. Broad-spectrum antibiotics (Cephalosporine and Metronidazole) were administered at induction of anesthesia. Patients were commenced on postoperative total parenteral nutrition (TPN) on the day after surgery and continued until gut function returned.

### Perioperative management in Aarhus

All patients underwent a CT of the thorax, abdomen and pelvis and a colonoscopy, unless already performed within the last month. Selected patients were evaluated by a diagnostic laparoscopy to clarify the extent of peritoneal malignancy or to obtain a biopsy. All patients were evaluated by a multidisciplinary team prior to CRS and HIPEC. Patients were fasted (no oral solids for 6 h and oral liquids allowed up to 2 h prior to induction of anesthesia). All patients had compression stockings and prophylactic thromboembolic treatment in the form of low-molecular-weight heparin. At commencement of anesthesia all patients received prophylactic antibiotics (Cephalosporine and Metronidazole). All patients received a nasojejunal tube intraoperatively. Bowel preparation was not used routinely.

To assess any postoperative complications patients were scheduled for intensive care admission for a minimum of 24 h followed by hospitalization in the surgical ward. All patients were treated according to the principles of ERAS protocols. Pain relief was managed using paracetamol intravenously and a thoracic epidural analgesic infusion of bupivacaine 1 mg/mL, fentanyl 2 µg/mL and adrenaline 2 µg/mL [26]. When gastrointestinal function was regained pain relief was changed to peroral paracetamol, non-steroid anti-inflammatory drugs (NSAID) and morphine as necessary. Antibiotic prophylaxis was given from surgery to postoperative day (POD) 3 (Cephalosporine and Metronidazole). Patients were evaluated postoperatively with daily blood tests from POD1 to POD3. Following POD3, the blood tests were repeated every third day until discharge or transfer to another hospital.

A regime of postoperative enteral nutrition (EN) via a nasojejunal tube was followed. The total daily energy expenditure (TDEE) was calculated and EN (Nutrison Protein Plus, Nutricia, Amsterdam, The Netherlands) administered as 50% of TDEE on POD1, 75% of TDEE on POD2 and 100% of TDEE on POD3.

Removal of the tube was at the discretion of the surgeon based on evaluation of nausea, vomiting, aspiration and ability to tolerate an oral diet.

Patients were discharged or transferred to a local hospital when the following criteria were met; no earlier than POD7, sufficient pain management on oral analgesics, tolerance of an oral diet (both liquids and solid food), recovery of gastrointestinal function and unremarkable blood results.

### Outcomes

The primary outcome was incidence and duration of PPOI, defined as more than five days from surgery without defecation [8, 22]. All patients reported parameters of postoperative gastrointestinal function in a diary. From the day of surgery to POD10 or, if earlier, to the day of discharge, the patient reported the following parameters; nausea, vomiting, ability to tolerate an oral diet, passage of flatus, passage of stool, having a nasogastric/-jejunal tube, having to reinsert a nasogastric tube and receiving enteral and/or parenteral nutrition. The secondary outcomes were, days with nausea and/or vomiting. Time to removal of nasogastric/nasojejunal tube was used as a measure of gastric paresis. Time to tolerance of an oral diet and postoperative complications were also secondary outcomes.

All patient records were investigated for postoperative complications using the Peritoneal Malignancy Postoperative Complications Proforma and graded according to the Clavien-Dindo classification [27].

### Statistical analysis

Continuous variables are presented as median (range) and tested with Mann–Whitney U test. Categorical variables are presented as proportions. Distributions are compared using Kolmogorov–Smirnov test. A linear regression analysis is performed to test associations between continuous outcomes. Patients are divided into two groups for statistical analyses; a group of PMP patients and a group including colorectal cancer, appendix cancer, goblet cell carcinoma, mesothelioma and ovarian cancer. These groups are for practical reasons termed “PMP” and “Other”. All statistical tests use a type I error set at α 0.05. A two-sided p-value of <0.05 was considered statistically significant. Statistical software XLSTAT (Addinsoft, 2019; XLSTAT Statistical and Data Analysis Solution, Long Island, NY, USA) and STATA/SE v14.2 (StataCorp, Texas, USA) were used for analyses.

### Ethics

The patients were given oral information by a trained health care professional. Informed consent was given on a voluntary basis and could be withdrawn at any time without having any impact on current or future treatment. This study complies with the relevant national regulations, institutional policies and in accordance the tenets of the Helsinki Declaration.

## Results

A total of 85 patients were included in the study (Aarhus: 21, Basingstoke: 64). Forty-nine of the patients were males. Perioperative characteristics are given in Table 1. Complete cytoreduction was achieved in 82 patients (96%) and HIPEC was performed in all patients. All diaries (n=85) were returned completed at follow-up with no data-loss. Data obtained from patient diaries are presented in Table 2.

**Table 1:** Perioperative data.

	PMP, n=38	Other^a^, n=47	Total, n=85	p-Value
Gender, male/female	19/19	30/17	49/36	0.270
Age, median (range)	54 (32–83)	58 (27–82)	58 (27–83)	0.253
ASA score, 1/2/3	2/22/14	4/37/6	6/59/20	0.011
BMI, median (range)	27.2 (19.4–36.3)	26.5 (17.5–35.3)	26.8 (17.5–36.3)	0.485
Center, Aarhus/Basingstoke	3/35	18/29	21/64	0.002
PCI score, median (range)	27 (3–39)	6 (0–39)	12.5 (0–39)	<0.001
Complete CRS, yes/no	35/3	47/0	82/3	0.086
Duration of surgery, h	8.3 (4.5–13.0)	5.6 (2.5–11.2)	7.0 (2.5–13.0)	<0.001

PMP, pseudomyxoma peritonei; ASA, American Society of Anesthesiologists; BMI, body mass index; PCI, Peritoneal Cancer Index; CRS, cytoreductive surgery. ^a^Other diagnoses include colorectal cancer, appendix cancer, goblet cell carcinoma, mesothelioma and ovarian cancer. Continuous variables are presented as median (range) and tested with Mann–Whitney U test. Categorical variables are presented as proportions and tested using Fisher’s test. Distributions were compared using Kolmogorov–Smirnov test.

**Table 2:** Postoperative gastrointestinal function.

	PMP, n=38	Other^a^, n=47	Total, n=85	p-Value
Days to flatus, median (range)	5 (1–12)	4 (1–10)	4 (1–12)	0.011
Days to defecation, median (range)	7.5 (1–14)	5 (1–13)	6 (1–14)	0.018
Days with nasogastric/nasojejunal tube, median (range)	8 (1–49)	6 (1–15)	7 (1–49)	0.019
Placing of new nasogastric/nasojejunal tube, yes/no	9/29	5/42	14/71	0.144
Enteral nutrition, n	5	17	22	0.024
Duration of enteral nutrition, median (range)	0 (0–29)	0 (0–6)	0 (0–29)	0.039
Parenteral nutrition, n	36	31	67	0.001
Duration of parenteral nutrition, median (range)	10 (0–46)	7 (0–16)	8 (0–46)	<0.001
Time to tolerance of oral diet, median (range)	8.5 (4–11)	6 (1–11)	7 (1–11)	<0.001
Days with nausea, median (range)	2 (0–8)	2 (0–9)	2 (0–9)	0.687
Days with vomiting, median (range)	0 (0–5)	1 (0–5)	0 (0–5)	0.021

PMP, pseudomyxoma peritonei. ^a^Other diagnoses include colorectal cancer, appendix cancer, goblet cell carcinoma, mesothelioma and ovarian cancer. Continuous variables are presented as median (range) and tested with Mann–Whitney U test. Categorical variables are presented as proportions and tested using Fisher’s test.

Forty-six patients (54%) developed PPOI. The incidence of PPOI was similar between the two centers (Aarhus: 10, Basingstoke: 36, p=0.62).

We found no significant differences between the two centers with regard to age (p=0.139), ASA score (p=0.162), BMI (p=0.289) or time to first defecation (p=0.313). A significantly larger proportion of patients in Basingstoke were diagnosed with PMP compared to Aarhus (p=0.002). The PCI scores were also significantly higher in Basingstoke (p=0.011); however, this was not the case when analyzing PMP-patients separately (PMP: p=0.763, Other: p=0.512). As a result, we present data from the two centers merged.

In general, PMP-patients had higher PCI scores than all other patients (p<0.0001), and increasing PCI score correlated with longer durations of surgery, p<0.0001.

The risk of PPOI did not correlate to gender (p=0.052), increasing BMI (p=0.588), higher ASA-score (p=0.371) or longer duration of surgery (p=0.688). Being diagnosed with PMP did not increase the risk of developing PPOI (p=0.079). Increasing PCI-score did not increase the risk of developing PPOI (p=0.389).

### Postoperative gastrointestinal function

**Figure 1:**
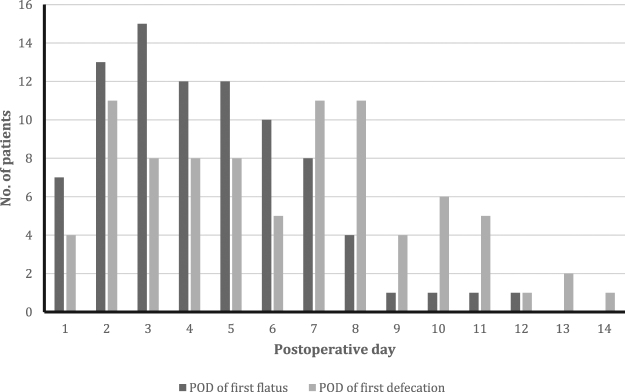
Histogram presenting the distribution of time to first flatus and first defecation in all patients.

Table 2 presents an overview of parameters on postoperative gastrointestinal function. Median time to first flatus was 4 days (range 1–12), with PMP patients having significant longer times to first flatus compared to other patients, p=0.011. Increasing PCI-score did not correlate to longer time to first flatus (p=0.107). Median time to first defecation was 6 days (1–14) with PMP patients having longer time to first defecation compared to other patients, p=0.018. Figure 1 presents a histogram of the distribution of time to first flatus and first defecation. Increasing PCI-score did not correlate to longer time to first defecation (p=0.11).

Median time to removal of nasogastric or nasojejunal tube was 7 days (range: 1–49). PMP patients had a nasogastric or nasojejunal tube for longer than the rest of the patients (p=0.019). Increasing PCI-score correlated to longer time to removal of nasogastric or nasojejunal tube, p=0.002.

Patients with PPOI had longer time to first flatus (p<0.001) and longer time to removal of nasogastric or nasojejunal tube (p=0.001). Fourteen patients had a nasogastric/nasojejunal tube replaced after removal of the first tube. PPOI did not result in increased risk of having tube replacement after removal of the first tube (p=0.776).

Three patients (14.3%) in Aarhus were moved from enteral to parenteral nutrition, all after developing PPOI.

Median time to tolerance of an oral diet was 7 days (range: 1–11). Time to tolerance of an oral diet correlated to time of first defecation (p=0.002) and time to removal of tube (p<0.001). Patients with PPOI had significantly longer time to tolerance of an oral diet (p=0.008). Increasing PCI-score correlated with longer times to tolerance of an oral diet (p<0.0001).

Sixty-five patients (76.5%) reported postoperative nausea for a median of 2 days (range: 0–9). Forty patients (47%) reported postoperative vomiting for a median of 0 days (range: 0–5). We found no difference between the two patient groups with regards to days with nausea (p=0.687), but PMP patients had more days with vomiting compared to the other patients (p=0.021). Development of PPOI was not associated with an increase in days with nausea (p=0.386) or vomiting (p=0.704).

**Figure 2:**
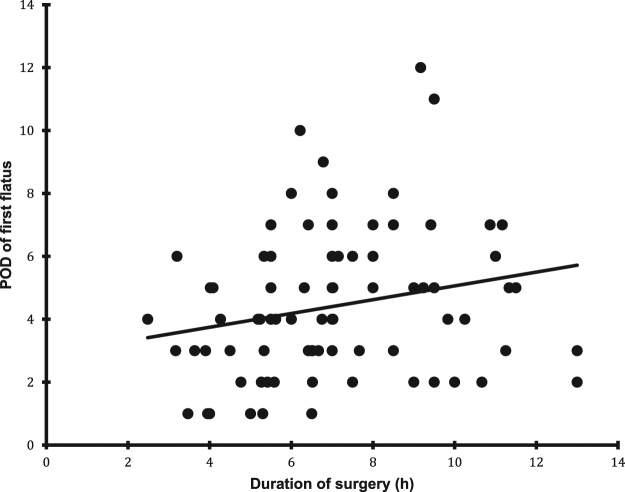
The relation between duration of surgery and time to first flatus (p=0.015).

**Figure 3:**
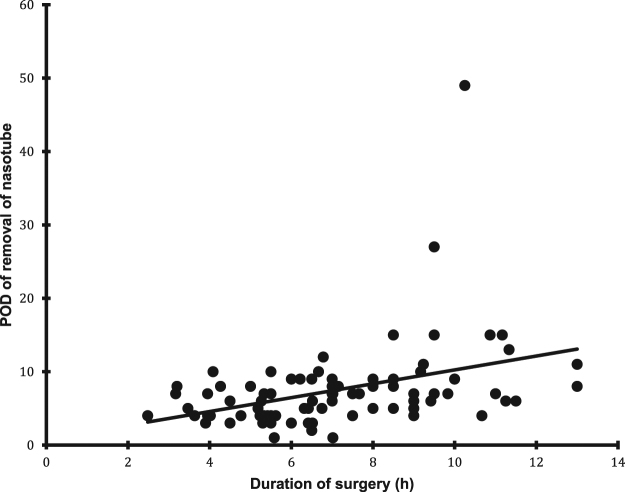
The relation between duration of surgery and time removal of nasogastric or nasojejunal tube (p<0.0001).

**Figure 4:**
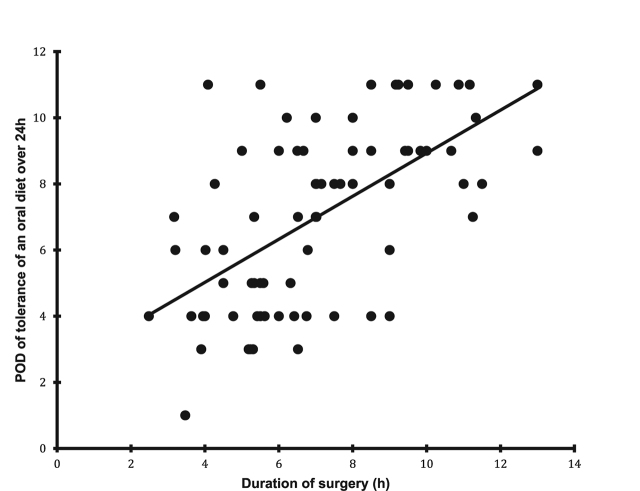
The relation between duration of surgery and time to tolerance of an oral diet (p<0.0001).

We found a correlation between duration of surgery and time to flatus (Figure 2), removal of nasogastric or nasojejunal tube (Figure 3) and time to tolerance of an oral diet (Figure 4) but not with time to defecation.

### Postoperative complications

Table 3 presents an overview of postoperative complications in patients with or without PPOI Thirty-five patients with PPOI had postoperative complications compared to 27 of the patients without PPOI (p=0.625). The postoperative complications were of similar severity between the PPOI-group and no-PPOI-group (p=0.276).

**Table 3:** Postoperative complications.

	PPOI, n=46 (54%)	No PPOI, n=39	Total, n=85	p-Value
No complications, n	11	12	23	0.625
Highest complication grade^a^, 0/1/2/3	11/9/25/1	12/2/19/6	23/11/44/7	0.276
Pneumonia, n	7	3	10	0.331
Pleural effusion, n	7	2	9	0.170
Urinary tract infection (UTI), n	1	2	3	0.591
Wound infection, n	3	3	6	1.000
Wound dehiscence, n	8	5	13	0.764
Intraabdominal infection, n	5	4	9	1.000
Gastrointestinal perforation, n	0	1	1	NA
Intraabdominal bleeding, n	1	1	2	1.000
Need for transfusion of blood components, n	9	7	16	1.000
Thrombocytosis, n	6	4	10	0.748
Cardiac arrhythmia, n	3	3	6	1.000
Pulmonary embolism, n	1	1	2	1.000
Deep venous thrombosis (DVT), n	1	2	3	0.591

PPOI, prolonged postoperative ileus. ^a^If developing multiple postoperative complications, the highest of the complication grades were registered. Categorical variables are presented as proportions and tested using Fisher’s test. Distributions were compared using Kolmogorov–Smirnov test.

Frequent postoperative complications included need for transfusion of blood components (19%), wound dehiscence (15%), pneumonia (12%) and thrombocytosis (12%). Development of POI did not increase the risk of any specific postoperative complications. The risk of postoperative pneumonia was not different between patients receiving postoperative enteral or parenteral nutrition (p=1.000).

## Discussion

This study shows that, despite increased efforts on implementation of enhanced recovery after surgery programs aiming to reduce PPOI, a significant proportion of patients develop PPOI after CRS and HIPEC. In this study, more than half of the patients developed PPOI. There was a significant relationship between duration of surgery and gastrointestinal function (time to first flatus and time to removal of nasogastric or nasojejunal tube). The relationship between longer operation time and PPOI has been described previously [28, 29]. Obviously, shorter operative times might be beneficial to improve gastrointestinal function; however, a focus on shortening operative times might be hazardous.

In general, postoperative outcomes in colorectal surgery have improved with the introduction of ERAS programs [17, 18, 19, 20]. Although no major breakthroughs in the treatment of POI have been made, methods to reduce POI and PPOI are of increased interest for many clinical professionals. Thus, for example, opioids have been largely replaced by the use of epidural anesthesia and NSAID [11, 12, 19, 20]. Evidence also suggests that early oral feeding and early mobilization can reduce the incidence and extent of POI, in combination with the use of laxatives and anti-emetic agents [11, 12, 19, 20].

The methodological strengths of this study include the prospective design with registration of clearly defined clinical outcomes. The patient diary design, with patients instructed to register the presence of nausea or vomiting, passage of flatus and defecation, and consumption of a regular oral diet on a daily basis, helps to secure precise recording of data. The recording of exact times of first onset of these events by patients themselves, with the nursing staff and/or local investigators checking the completion of diary registration on a daily basis helps ensure accuracy of the dataset.

The limitations of this study include the confounding factor of the absence of standardization between the participating centers. In particular, the difference between an enteral nutrition-based strategy in Aarhus and a parenteral nutrition-based strategy in Basingstoke. We also saw a difference in patient group diagnoses between centers. However, the PCI scores were similar between centers when analyzing PMP-patients separately.

An inconsistency in the definition of POI in clinical trials has made it difficult to estimate the incidence of POI. Vather et al. conducted a systematic review and global survey on the definition of POI and Prolonged POI [30]. They recommended that POI was defined as passage of flatus or stool and tolerance of an oral diet before POD4. The definition of PPOI is more complex and defined as two or more of the following criteria on or after POD4; nausea or vomiting, inability to tolerate an oral diet, absence of flatus over the previous 24 h, abdominal distension or radiologic confirmation of POI. This definition was used by Peters et al. in the SANICS-II trial, investigated the effect of perioperative enteral nutrition on POI in patients undergoing segmental colonic resection [31]. They reported that the incidence of POI and PPOI to be 24.9% and 12.5%, respectively. We defined PPOI as first passage of stool after POD5 as a simpler definition [22, 23]. The time to first defecation can be compared between studies being median 2 days in the SANICS-II trial compared to median 6 days in our study. A retrospective study of 89 patients with advanced cancer operated on by open abdominoperineal excision and transpelvic vertical rectus abdominal musculocutaneous (VRAM)-flap found that 28% of the patients experienced PPOI [23]. This indicates a correlation between the amount of surgical trauma and intestinal manipulation on the risk of developing PPOI. The effects and pathogenesis of intraperitoneal chemotherapy on gastrointestinal motility is poorly documented but might partly account for the difference in PPOI between VRAM-patients (operating times median 5 h) and the current series of patients undergoing CRS and HIPEC-patients (operating times median 7 h).

As shown in this study, delayed gastric emptying is a common complication of uncertain origin after CRS and HIPEC. A Dutch randomized study of 42 patients undergoing CRS and HIPEC assessed if preservation of the right gastro-epiploic artery during standard omentectomy had a positive effect on gastric emptying after CRS and HIPEC [32]. However, no difference was noted between preservation of the gastro-epiploic artery or not during omentectomy on gastric emptying after CRS and HIPEC. They concluded that the extensive intestinal manipulation or the HIPEC were more likely causes of the delayed gastric emptying. This might also explain the positive correlation between duration of surgery and time to removal of nasogastric or nasojejunal tube found in this study. Data from Kalff et al. suggests that the degree of stomach paralysis or delayed gastric emptying correlates to the degree of manipulation of the stomach during surgery [33]. The use of a multi-lumen tube for both enteral nutrition via a jejunal limb and aspiration of gastric content via a gastric lumen requires further manipulation of the stomach compared to a single lumen nasogastric tube for aspiration of gastric content and might address the issue of PPOI. Dineen et al. investigated if placement of a feeding tube during CRS and HIPEC is of benefit in regard to improving postoperative nutrition [34]. They found feeding tube placement to be related to higher readmission rates and longer length of stay. However, the placement of feeding tubes was at the discretion of the attending physician based on the degree of suspicion for a prolonged ileus. This makes the findings questionable with suspicion of possible confounding by indication.

Several studies have reported that laparoscopic surgery shortens hospital stay in colorectal surgery [19]. However, open surgery is still required in patients with peritoneal malignancy [21]. Attempts to treat peritoneal malignancy by a laparoscopic technique or a combined laparoscopic and open technique may be sub-optimal and hazardous, since proper exposure is required, particularly to assess the peritoneal surfaces of the subphrenic spaces and the pelvis. The use of a combined technique might be feasible in highly selected cases and might result in lower rates of PPOI.

Ten patients (12%) developed postoperative pneumonia, a considerately higher proportion compared with patients operated on for colorectal cancer [31]. Neither PPOI nor the use of enteral nutrition compared with parenteral nutrition had an effect on the risk of pneumonia. Peters et al. found a trend towards a higher incidence of pneumonia in patients receiving post-pyloric enteral nutrition, questioning the safety of perioperative feeding [31]. In difference, they started enteral nutrition 3 h prior to surgery (perioperative regime) compared to the postoperative regime of enteral nutrition in Aarhus.

The European Society for Parenteral and Enteral Nutrition (ESPEN) Guidelines recommend early tube feeding (within 24 h) to be initiated in patients undergoing gastrointestinal surgery for cancer where early oral nutrition cannot be started, and in those in whom oral intake will be inadequate (<50%) for more than 7 days [35]. The evidence for nutritional therapy interventions in surgical patients has been critically assessed in several meta-analyses and Cochrane Database Systematic Reviews [36, 37]. However, there is considerable inconsistency and the evidence is still unconvincing with regards to the effect of TPN in comparison with oral/enteral nutrition. Focusing on patients after gastrointestinal surgery, Mazaki et al. found beneficial effects of enteral nutrition on anastomotic leaks and shorter hospital length of stay [38]. Zhao et al. found a shorter time to flatus, shorter hospital length of stay and a greater increase in albumin levels postoperatively in patients treated with enteral nutrition [39]. However, no significant difference on postoperative mortality was reported.

There have been reports suggesting other beneficial effects of enteral nutrition in addition to delivering nutrients. Mucosal structural changes occur in the absence of enteral nutrition, leading to impairment in the function of the gastrointestinal barrier. Such impairment stimulates migration of bacteria from the intestinal lumen into the submucosal tissue. This migration triggers epithelial inflammation facilitating release of proinflammatory and anti-inflammatory factors, leading to further degradation of gastrointestinal mucosal resistance. The physiological stimulus of enteral nutrition helps to maintain gastrointestinal function and motility since the nutrients stimulate the secretion of motility-regulating gastrointestinal hormones [40]. Several studies have investigated the effect of enteral nutrition on gastrointestinal inflammation through vagal stimulation in patients intolerant to an oral diet. The SANICS-II trial by Peters et al. did not find a clinically significant effect on POI and anastomotic leakage [31]. However, a previous trial (SANICS-I) showed that early commencement of enteral nutrition after open rectal surgery reduced POI [24].

## Conclusions

This study reports that a significant number of patients develop PPOI following CRS and HIPEC despite the implementation of early enhanced recovery protocols. New regimens for preventing and treating PPOI is of high clinical value and further research on the prevention and treatment of PPOI in extensive abdominal surgery is important.
